# Effect of imidacloprid ingestion on immune responses to porcine reproductive and respiratory syndrome virus

**DOI:** 10.1038/s41598-018-30093-6

**Published:** 2018-08-02

**Authors:** J. Hernandez, A. Volland, B. J. Leyshon, M. Juda, J. M. Ridlon, R. W. Johnson, A. J. Steelman

**Affiliations:** 1 Department of Animal Sciences, University of Illinois Urbana-Champaign, Urbana, IL 61801 USA; 2 Integrative Immunology and Behavior Program, University of Illinois Urbana-Champaign, Urbana, IL 61801 USA; 3 Carl R. Woese Institute for Genome Biology, University of Illinois Urbana-Champaign, Urbana, IL 61801 USA; 4 Division of Nutritional Sciences, University of Illinois Urbana-Champaign, Urbana, IL 61801 USA; 5 Department of Neuroscience, University of Illinois Urbana-Champaign, Urbana, IL 61801 USA

## Abstract

Nicotine and acetylcholine cause immunosuppresion by signaling to the α7 nicotinic acetylcholine receptor (α7 nAChR) on immune cells. Neonicotinoids are nAChR agonists and widly used insecticides. We aimed to define the immunosuppressive potential of dietary exposure to the neonicotinoid imidacloprid (IMI) on the generation of innate and adaptive immune responses to porcine reproductive and respiratory syndrome virus (PRRSV). Piglets were randomized into groups based on diet and infection. Behavioral signs of illness were recorded. Urine IMI levels were measured by high performance liquid chromatography-mass spectrometry. Flow cytometry was used to determine the expression pattern of the α7 nAChR on porcine leukocytes as well as the effects of infection and treatment on circulating leukocyte populations. Serum cytokines and PRRSV-specific antibody levels were determined by ELISA. Viral RNA in lung, spleen and plasma was determined by RT-qPCR. Pigs in the treatment group had elevated urine levels of IMI. Treatment with IMI reduced body weight, caused bouts of hypothermia, increased serum IL-10 and elevated levels of virus-specific antibodies. Viral RNA levels in the spleen showed a trend toward being increased in pigs fed IMI. Our data indicates that IMI injection may modulate virus specific immune function during PRRSV infection.

## Introduction

Neonicotinoids are a relatively new class of insecticides that were initially developed during the late 1980’s^1^. The first neonicotinoid, imidacloprid, was introduced as an insecticide in 1991 and is registered for over 140 crop uses in greater than 120 countries making it by far the most commonly used insecticide in the world^2^. Subsequently, the neonicotinoids thiamethoxam, clothianidin, thiacloprid, acetamiprid, nitenpyram and dinotefuran were introduced into the agricultural market as effective insecticides and, in terms of market share, rival imidaclopird^1^. The wide-spread use of neonicotinoids as insecticides gives credence to their effectiveness which is undoubtedly attributable to their environmental stability, water solubility, as well as their ability to be rapidly absorbed through roots, foliage, pollen and fruit and disseminated throughout the entire plant anatomy^3^. In fact, the efficiency by which the neonicotinoids imidacloprid and clothianidin are systemically disseminated enables seed coating (i.e. imidaclopird; 1 mg/seed) as an effective treatment strategy. Foliar spray application, trunk injection and drip-irrigation are also an effective means of treating agricultural crops with neonicotinoids^4^.

In the U.S., it has been estimated that 900 tons of clothianidin are applied annually to 46 million acres, with corn accounting for 95% of this usage. The annual use of imidacloprid is estimated at 890 tons, of which 60% is used on soybean and cotton^1^. Presently, almost all corn seeds in the U.S. are preemptively coated with neonicotinoids prior to planting. This particular practice has raised some concerns for the following reasons: First, because of their environmental stability, residual neonicotinoids become elevated in agricultural fields during cultivation and remain elevated following harvest^5^. Secondly, neonicotinoid levels are increased in water sources that surround treated areas^6^. One month after of planting, puddles in corn fields were shown to contain up to 2.3 µg/L of clothianidin^7^. Streams located in corn and soybean producing regions of the U.S. have been shown to contain concentrations as high as 0.5 µg/L^8^, and in Canada as much as 3.1 µg/L clothianidin^9^. Because the half-life of clothianidin in soil can exceed 1000 days^6^ and water treatment does not alter neonicotinoid levels^10^, the continued use of this neonicotinoid as a seed coating agent would be expected to increase residues in ground water. Data also suggests that neonicotinoids are detectable in food. For instance, corn kernels at harvest contain imidacloprid at concentrations at or below 1 µg/kg^11^ and could be detected in cereals post processing^12^. A recent study revealed that a large percentage of commercially available produce and honey contained at least one neonicotinoid and in some cases relatively high concentrations (i.e. 100 ng/g apple)^13,14^. Notably, when ingested, neonicotinoids are absorbed in the intestine and disseminated to other organs^15,16^. Human consumption is further evidenced by mass-spectroscopy analysis of urine samples. In particular toxicological modeling after toxicokinetic analysis of ingested deuterated neonicotinoids indicated that urine levels of imidacloprid and clothianidin, were correlated with the consumption of fruits and vegetables^17^. In children aged three years, urine neonicotinoids were highest in the summer months^18^.

Neonicotinoids are nicotine analogues that function as nicotinic acetylcholine receptor (nAChR) agonists by binding to the insect nAChR^19^. Acetylcholine receptors are ligand-gated ion channels that are constitutively expressed in the central nervous system (CNS), muscle tissue, and cells of the immune system. Like nicotine, neonicotinoids exert their toxicity to insects in a manner that is dependent on the constitutive activation of nAChRs that are highly expressed throughout the insect nervous system, leading to energy store depletion and death. However, the activation of insect nAChRs by neonicotinoids are orders of magnitude greater than that of nicotine^3^. The decreased affinity of neonicotinoids for α4β2 nAChR and α7 nAChRs, which are abundantly expressed in the CNS, likely explains their reduced neurotoxic potential in mammals^3^.

Communication between the brain and the immune system is required to efficiently resolve inflammatory responses. The cholinergic anti-inflammatory pathway (CAP) is partially responsible for fulfilling this function. Activation of this pathway is initiated when efferent neuron depolarization causes post-synaptic secretion of acetylcholine in the liver and intestine or depolarizes the splenic nerve. The latter results in norepinephrine release within the spleen which signals to β2-andrenergic receptors (β2AR) on a subset of T-cells^20^. In response to this signal β2AR^+^ T-cells secrete acetylcholine. Acetylcholine-mediated activation of α7 nAChRs on macrophages and dendritic cells activates the JAK2/STAT3 pathway, inhibits NF-kB signaling and decreases proinflammatory cytokine secretion^21^. The immunosuppressive capability of the CAP is illustrated by the fact that experimental manipulation of this pathway greatly influences the severity of shock and inflammation induced tissue injury^22–24^. In a similar fashion, nicotine can suppress proinflammatory responses that occur during viral infection or in models of autoimmunity and does so in a manner that is thought to be contingent on the activation of α7 nAChRs on immune cells^25,26^.

Since neonicotinoids were shown to signal to mammalian nAChRs, neonicotinoids might activate the cholinergic anti-inflammatory pathway and promote immunosuppression. To date, the effect of either acute or chronic neonicotinoid exposure on immune function remains obscure. In fact, few studies provide data on the topic. First, in honey bees sub-toxic exposure to the neonicotinoids imidacloprid and clothianidin caused substantial NF-κB suppression and resulted in increased susceptibility to deformed wing virus, a pathogen associated with colony collapse disorder^27^. Similarly, clothianidin treatment of human THP-1 cells suppressed NF-κB activation and inhibited TNF production after an LPS challenge^28^. Prenatal exposure to imidacloprid in birds resulted in T-cell immunosuppression in chicks^29^. Finally, high dose oral exposure to acetamiprid in rats decreased peritoneal macrophage nitrite production following LPS stimulation^30^. The ability for low-dose neonicotinoid exposure to alter immune response generation may have implications for human and agricultural animal health. Using a neonatal piglet model, we questioned whether dietary exposure to the neonicotinoid imidacloprid was capable of altering the generation of innate and adaptive immune responses to porcine reproductive and respiratory syndrome virus (PRRSV). Infection of swine with PRRSV incurs over $600 million in annual losses to the pork industry and therefore represents a particularly relevant pathogen to the agricultural industry.

## Materials and Methods

### Animal Husbandry

All of the experimental procedures described herein were approved by the Institutional Animal Care and Use Committee at the University of Illinois Urbana-Champaign and were performed in accordance with guidelines of the National Institutes of Health.

Eight to twelve week old male and female C57BL/6 J mice were used for these studies. All mice were originally obtained from Jackson Laboratory (Cat. No. 000664; Bar Harbor, ME). Mice were housed under constant 12-h light/dark cycles and constant temperature in covered cages and fed with a standard rodent diet ad libitum.

Naturally farrowed male and female domestic Yorkshire piglets were obtained from the University of Illinois swine herd at postnatal day 2 or 3. Prior to arrival, all piglets received 1 ml of iron dextran (200 mg/ml), and antibiotics (Excede for swine; Zoetis, Parsippany, New Jersey) by intramuscular injection. Upon arrival, pigs received an additional dose of penicillin (60 kU/pig, Butler Schein Animal Health, Dublin, OH). Piglets were weight matched into one of four possible treatments in a 2 by 2 factorial arrangement, imidacloprid supplementation and PRRSV infection. Piglets were allowed 5 days of acclimation to their new environment prior to experimentation. All piglets were housed in ABSL2 containment, with PRRSVs inoculated piglets housed separately from saline inoculated controls in order to avoid cross-contamination. All piglets were housed individually to control for food intake. Each cage contained individual Lectro-kennel heated pads (K&H Manufacturing; Colorado Springs, CO) to help maintain appropriate local temperatures as well as a toy for environmental enrichment (plastic Jingle Ball™, Bio-Serv, Frenchtown, NJ). The cage flooring consisted of rubber coated metal (Tenderfoot/NSR, Tandem Products, Inc., Minneapolis, MN) that allowed urine and feces to fall through the flooring.

### Isolation of splenocytes

Splenocytes were isolated from mice as described previously^31^. In brief, animals were anesthetized by intraperitoneal (i.p.) injection of ketamine (85–100 mg/kg) and xylazine (10–13 mg/kg) diluted in 0.1 ml of phosphate buffered saline (PBS; pH 7.4) and spleens were removed, broken apart with the plunger from a 3 ml syringe and passed through a 70 μm cell strainer (Corning, Corning NY). The strainer was washed with RPMI 1640 media (UIUC cell media facility, Urbana, IL) supplemented with L-glutamine (1 mM) (Sigma Aldrich, St. Louis, MO), sodium pyruvate (1 mM) (Sigma Aldrich, St. Louis, MO), 10% heat inactivated fetal bovine serum (FBS) (Corning, Corning, NY) and 1% penicillin/streptomycin (100 U each, Thermofisher, Carlsbad, CA) (R10S media). Cells were centrifuged at 300 × *g* for 7 min., and pellets were lysed with 2 ml of red blood cell (RBC) lysis buffer (Biolegend; San Diego, CA) for 1–2 min. at room temperature. The cells were washed with R10S and made into a single cell suspension in R10S before being counted using a hemocytometer.

### Dietary imidacloprid treatment and PRRSV infection

Piglets were limit fed a Liqui-wean milk replacer diet (Milk Specialties; Eden Prairie, MN) that was reconstituted daily to a final concentration of 206 g/L using tap water and supplied at a rate of 285 ml/kg/d delivered to a stainless steel bowl from a reservoir via a peristaltic pump (Control Company, Friendswood, TX) such that piglets received their daily allotted milk over the course of 14 meals (one per hour from 0600 hr to 2400 hr). However, to ensure that piglets consumed the treatment diet the first meal of each day contained either vehicle or IMI. Each day pigs in the treatment group received a diet supplemented with IMI (5 mg/kg/d; 95% purity, BOC Sciences, Shirley, NY) dissolved in dimethyl sulfoxide (DMSO) and diluted 1/1000 in milk replacer. Pigs in the non-treatment group received vehicle (DMSO diluted in milk replacer). Diets were freshly prepared each day. Dietary treatments for both groups began the day prior to infection and lasted the duration of the study.

The P129-BV strain of porcine reproductive respiratory syndrome virus (PRRSV) was kindly provided by Dr. William G. Van Alstine (School of Veterinary Medicine at Purdue University West Lafayette, Indiana). Between postnatal days 5–7 piglets were inoculated with either 1 ml of sterile PBS or PRRSV (1 × 10^5^ TCID_50_) dissolved in 1 ml sterile PBS.

### Observation

Weights, rectal temperatures, feeding scores, and sickness behaviors were recorded daily. Feeding scores were recorded to assess willingness of the piglets to consume their first daily meal using a scoring system where 1 = no attempt to consume the milk; 2 = attempted to consume the milk, but did not finish within 1 min.; 3 = consumed all of the milk within 1 min.

### Porcine euthanasia and tissue collection

Blood was collected on days 0, 3, 7, and 14 post infection (p.i.) via jugular venipuncture into K2 EDTA plasma tubes and uncoated serum tubes (BD; Franklin Lakes, New Jersey, USA). Serum was harvested by centrifugation at 2000 × *g* for 15 min. Peripheral blood mononuclear cells (PBMCs) were isolated from K2 EDTA coated tubes by Ficoll (Sigma; St Louis, MO) gradient centrifugation.

At the termination of the experiments piglets were anesthetized using a telazol:ketamine:xylazine drug cocktail [50 mg of tiletamine plus 50 mg of zolazepam reconstituted with 2.5 mL ketamine (100 g/L) and 2.5 mL xylazine (100 g/L); Fort Dodge Animal Health, Fort Dodge, IA] administered intramuscularly at 0.03 mL/kg body weight. Piglets were then euthanized via intracardiac injection of sodium pentobarbital (86 mg/kg body weight; Fatal Plus, Vortech Pharmaceuticals, Dearborn, MI). Lung and spleen biopsies were isolated at day 14 p.i. Single cell suspensions from spleen biopsies were prepared by passing tissues through 70 µm filters (Corning, Corning NY) into R10S. Cells were centrifuged at 300 × *g* for 5 min. and lysed with 3–5 ml of RBC lysis buffer for 7 min., then washed and re-suspend in R10S. Cells were counted using a hemocytometer under an inverted microscope.

Urine was extracted from the bladders piglets using a sterile 10 ml syringe and 21 g needle. After collection the urine was placed into sterile 15 ml conical tubes and stored at −80 °C until analysis.

### Urine neonicotinoid measurement by mass spectroscopy (LC-MS/MS)

The concentration of imidacloprid in urine was determined by mass spectroscopy. Isotope labeled d4-imidacloprid (Sigma Aldrich; St. Louis, MO) was prepared at 150 pg/μl in acetonitrile (ACN) and was used as an internal standard for imidacloprid measurements of treatment urine. Bond Elut PCX 30 mg polymeric strong cation exchange columns (Agilent Technologies; Santa Clara, CA) were purchased for solid phase extraction (SPE). Other reagents used for sample preparation and cleanup included LC-MS grade ACN, ammonia solution (7 M), phosphoric acid (2%), formic acid (2%), and HPLC grade methanol. The procedure for sample preparation and cleanup was adapted from Ueyama *et al*.^32^. In brief, 5 μl of treatment urine and 5 μl of 150 pg/μl imidacloprid-d4 was mixed with 990 μl water and 1 ml of phosphoric acid solution (2%). The mixture was incubated at 37 °C for 10 min. with gentle shaking in order to break up any urine crystals.

SPE was performed using a vacuum chamber. The SPE column was preconditioned using 500 μl of 5% ammonia solution in methanol combined with ACN (1:1. v/v) followed by 500 μl water. The 2 ml urine sample was then loaded on to the SPE column. The initial drop of solution was pulled through the column by vacuum, then gravity was used to elute the remaining sample. This was followed by 500 μl of formic acid solution (2%) and 3 min. of dry time using the vacuum. The final fraction was collected in a new tube and eluted drop-wise with gravity using 500 μl of methanol.

The final elution was evaporated under a stream of nitrogen at room temperature. The dry residues were sonicated for 10 min. after addition of 170 μl of ACN. This sample was centrifuged at 19,500 × *g* for 5 min. and the supernatant was used for LC-MS/MS analysis.

LC-MS/MS was performed on a Waters Acquity UPLC coupled with a Waters Synapt G2-Si ESI MS (Waters Co., Milford, MA, USA). Samples were separated on a Waters Cortecs UPLC C_18_ column (1.6 μm particle size) (2.1 mm × 50 mm) with a column temperature of 25 °C. The injection volume was 10 μl. The mobile phase consisted of Solvent A: H_2_O containing 5% ACN and 0.1% formic acid, and Solvent B: ACN containing 5% H_2_O and 0.1% formic acid. The initial mobile phase was 100% Solvent A. Over the next 2.5 minutes Solvent B was increased linearly until reaching 5% and was increased linearly again to 70% over the next 1.5 minute period. At 5 minutes, the mobile phase was 100% Solvent B before returning to the initial mobile phase over the next 2 minutes. The flow rate was 20 μl min^−1^ with a total run time of 7 min.

The MS/MS was operated with electrospray ionization (ESI) in positive ion mode using multiple reaction monitoring (MRM) in order to increase sensitivity. Nebulizer gas pressure (87 psi) was maintained at 600 °C and gas flow was 1000 L hour^−1^. High purity nitrogen was utilized as the collision cell gas and the capillary voltage was set at 3,000 V in positive mode. Raw chromatographs and mass spectrogram data were processed with MassLynx 4.1 Software (Waters). Peak area ratio of urinary imidacloprid to IS (d4-imidacloprid) was used for quantification.

### Flow cytometry

The following antibodies were used to analyze mouse splenocytes by flow cytometry: α-CD3-eFluor450 (clone: 17A2), α-CD4-AlexaFluor488 (clone: GK1.5) α-CD8-PE (clone: 3B5), α-CD19-PE (clone: eBio1D3), α-CD11b-AlexaFluor488 (clone: M1/70), α-CD11c-eFluor450 (clone: N418), α-NK1.1-PE (clone: PK136), and α-CD49b-FITC (clone: DX5) all from eBioscience (San Diego, CA). α-Bungarotoxin-AlexFluor647 (Thermofisher, Carlsbad, CA) was used to examine α7 nAChR expression. In brief, 5 × 10^5^ cells were stained for 20 min. in PBS containing 2% FBS (flow buffer). Cells were then centrifuged at 300 × *g* for 5 min. and washed with flow buffer. Immunophenotyping was determined using a LSRII Flow cytometer (BD, San Jose, CA). Gates were determined using unstained and single stained samples. Results were analyzed using FCS Express 6 Flow cytometry software (De Novo Software, Los Angeles, CA).

The following antibodies were used for immunophenotyping pig leukocytes by flow cytometry: α-CD3ε-PE (clone: BB23-8E6-8C8), α-CD3ε-PerCP-cy5.5 (clone: BB23-8E6-8C8), α-CD3ε-PerCP (clone: BB23-8E6-8C8), α-CD3ε-FITC (clone: BB23-8E6-8C8), α-CD4α-PE-cy7 (clone: 74-12-4), α-CD8α-AlexaFluor647 (clone: 76-2-11), α-CD8α-PE (clone: 76-2-11), α-CD5-FITC (clone: 1H6/8, all from BioRad; Hercules, CA), α-SLA Class II-FITC (clone: 2E9-13, antibodies online; Atlanta, GA), α-CD80-eFluor450 (clone: 16-10A1), α-CD172a-PE (clone: 74-22-15 A), α-IFN-γ-PerCp (clone: P2G10), α-IFN-γ-PE (clone: P2G10), and α-IFN-γ-PerCP-cy5.5 (clone:P2G10; all from eBioscience, San Diego, CA). α-Bungarotoxin-AlexFluor647 (Thermofisher, Carlsbad, CA) was used to assess expression of α7 nAChR. 4,6-Diamidino-2-phenylindole (DAPI), and fixable viability dye 780 (eBioscience, San Diego, CA) were used to assess viability. In brief, 5 × 10^5^ cells were blocked with α-mouse CD16/CD32 (clone: 93; eBioscience, San Diego, CA) for 10 min. then stained for 20 min. with antibody suspended in 0.1 ml of flow buffer on ice. Cells were then centrifuged at 300 × *g* for 5 min. and washed with flow buffer.

Intracellular cytokine staining (ICS) was performed using 1 × 10^6^ cells following four hours of treatment with Brefeldin A (BFA, eBioscience, San Diego, CA) or with Phorbol 12-myristate 13-acetate (PMA; 50 ng/ml), ionomycin (250 ng/ml; both from Sigma-Aldrich, St. Louis, MO) and BFA in R10S. Following stimulation cells were blocked and surface antigens stained as described above. Cells were then centrifuged at 300 × *g* for 5 min. and fixed with fixation buffer (eBioscience, San Diego, CA) for 20 min. at 24 °C. After washing twice with 1 ml of permeabilization buffer (eBioscience, San Diego, CA) intracellular stains were applied to cells for 20 min. After staining, cells were washed with permeabilization buffer and re-suspended in flow buffer. Data from samples were acquired on a LSRII Flow cytometer (BD Biosciences, Franklin Lakes, New Jersey, USA). Gates were determined using unstained and single stained samples from the same tissue. Results were analyzed using FCS Express 6 Flow cytometry software (De Novo Software, Los Angeles, CA).

### Cytokine Measurements

Serum concentrations of porcine IL-10 and TNF as well as plasma concentrations of IL-6 were determined by ELISAs according to manufacturer’s instructions (R&D Systems, Minneapolis, MN).

### Serum Virus-Specific Antibody Determination

Serum PRRSV-specific antibody levels were determined by ELISA according to the specifications of IDEXX (Westbrook, ME). Serial dilutions of day 14 samples were used to determine antibody titer. Plates were measured at 680 nm and optical density (O.D.) values were recorded.

### Viral RNA Levels

Viral RNA in the lung, spleen, and plasma was estimated by qRT-PCR. In brief, total RNA was isolated from lung and spleen biopsies using Tri Reagent (Sigma Aldritch, St. Louis, MO) according to the manufacturer’s instructions. The RNA quality and purity was determined using a NanoDrop spectrophotometer ND-1000 (Thermofisher, Carlsbad, CA). Total RNA was reverse transcribed to cDNA, amplified and semi-quantified using VETMAX™ NA and EU PRRSV reagents on a Step One Plus Real-Time PCR System according to the manufacturer’s instructions (Applied Biosystems, Austin, TX). Results are reported as fold change in ΔCT.

### Statistical analysis

All analysis was performed using GraphPad Prism version 7.00 for Windows (GraphPad Software, La Jolla, CA) or SAS™ software. Where appropriate three-way, two-way, and one-way ANOVA’s, t-tests, and Mantel-Cox and Bonferroni post hoc tests were used to determine significance. Statistical significance was set at p ≤ 0.05.

### Data availability

The data generated during and/or analysed during the current study are available from the corresponding author on reasonable request.

## Results

### Expression pattern of α7 nAChRs on porcine immune cells

**Figure 1:**
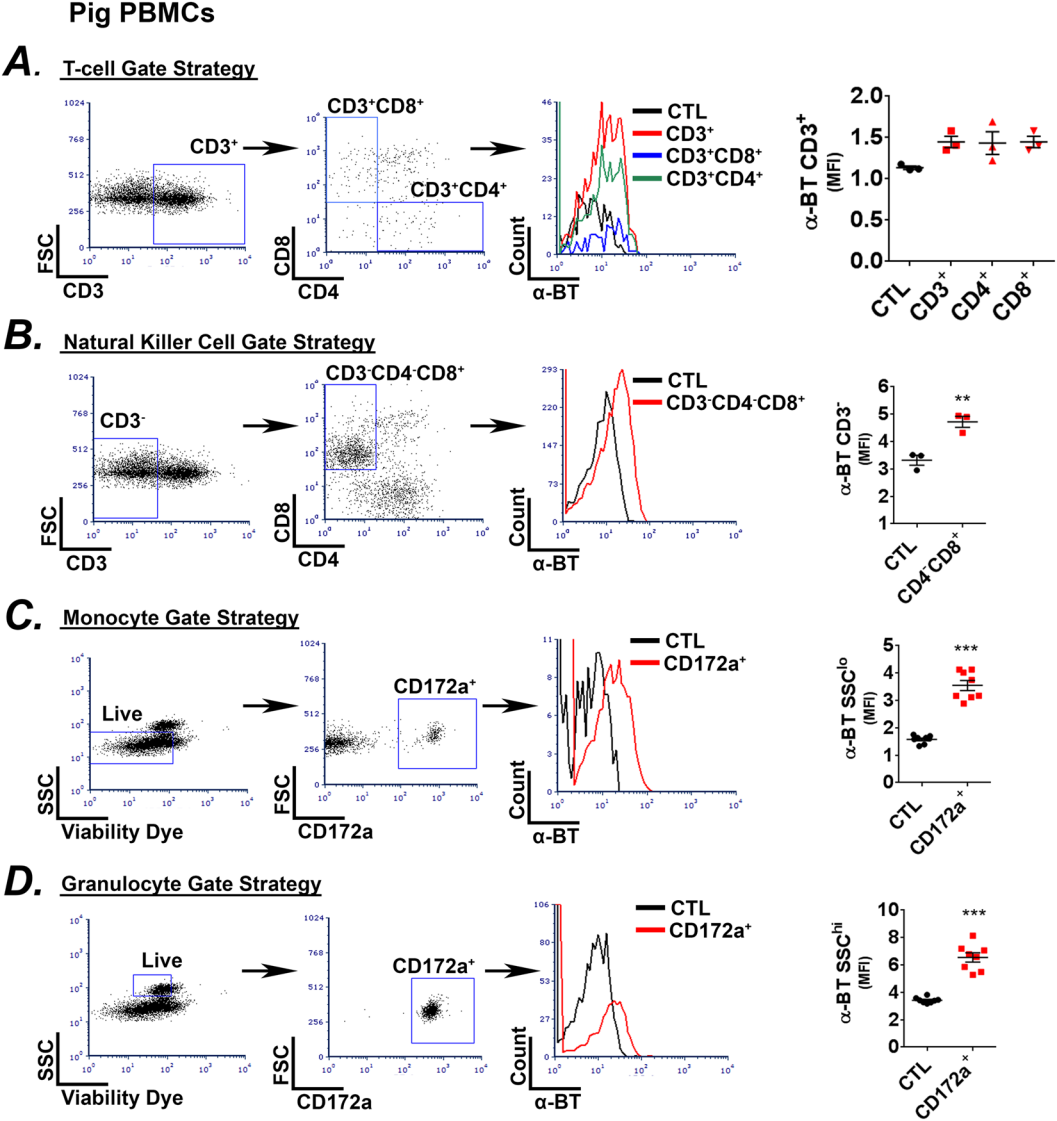
(**A**–**D**) The expression pattern of the α7 nicotinic acetylcholine receptor (α7nAChR) on porcine peripheral blood mononuclear cells (PBMCs) was analyzed using fluorescently labeled α-bungarotoxin (α-BT) by flow cytometry. (**A**–**D**) α-BT stain on T-cells (CD3^+^), helper T-cells (CD3^+^CD4^+^), and cytotoxic T-cells (CD3^+^CD8^+^) (**A**; n = 3), natural killer cells (CD3^−^CD4^−^CD8^+^) (**B**; n = 3), monocytes (CD172a^+^SSC^lo^) (**C**; n = 8), and granulocytes (CD172a^+^SSC^hi^) (**D**; n = 8) is shown. Flow cytometry gating strategy is provided for each cell type (*Left*), histograms show representative expression between unstained controls and α-BT stained cells (*Middle*), and graphs (*Right*) represent mean fluorescent intensity (MFI) of α-BT between unstained controls and α-BT stained cells. Results are means ± S.E. from 3–8 pigs. *p ≤ 0.05, ***p ≤ 0.001.

Both human and rodent immune cells express the alpha-7-nicotinic acetylcholine receptor (α7 nAChR) and signaling to these receptors dampens immune responses^23,24^. However, the expression profile of the α7 nAChR on porcine immune cells has not been defined. Therefore, we utilized fluorescently labeled α-bungarotoxin (α-BT), a neurotoxin that irreversibly and selectively binds α7 nAChR, and flow cytometry to determine the expression pattern. Mean fluorescent intensity analysis of α-BT stained cells isolated from blood indicated that both cytotoxic (CD3^+^CD8^+^) and helper (CD3^+^CD4^+^) T-cell populations exhibit a low level of receptor expression (Fig. 1A). In contrast, CD3^−^CD4^−^CD8^+^ natural killer cells (NK; Fig. 1B), CD172a^+^SSC^lo^ monocytes, and CD172a^+^SSC^hi^ granulocytes exhibited higher level of surface expression (Fig. 1C,D). Similar findings were obtained from splenocytes (Supplemental Fig. 1A,B). Interestingly, the expression profile of the α7 nAChR on porcine immune cells was nearly identical to that observed in mouse splenocytes (Supplemental Fig. 2A,B,D). An exceptional difference was that we did not observe α-BT staining on mouse NK cells (Supplemental Fig. 2D), a finding that has been reported previously^33^. These data indicate that the expression profile for the α7 nAChR on immune cells is relatively conserved across species and that the receptor expression pattern in piglets is more similar to that of humans^34^.

### Effects of infection and dietary imidacloprid on weights, temperatures and feeding scores

**Figure 2:**
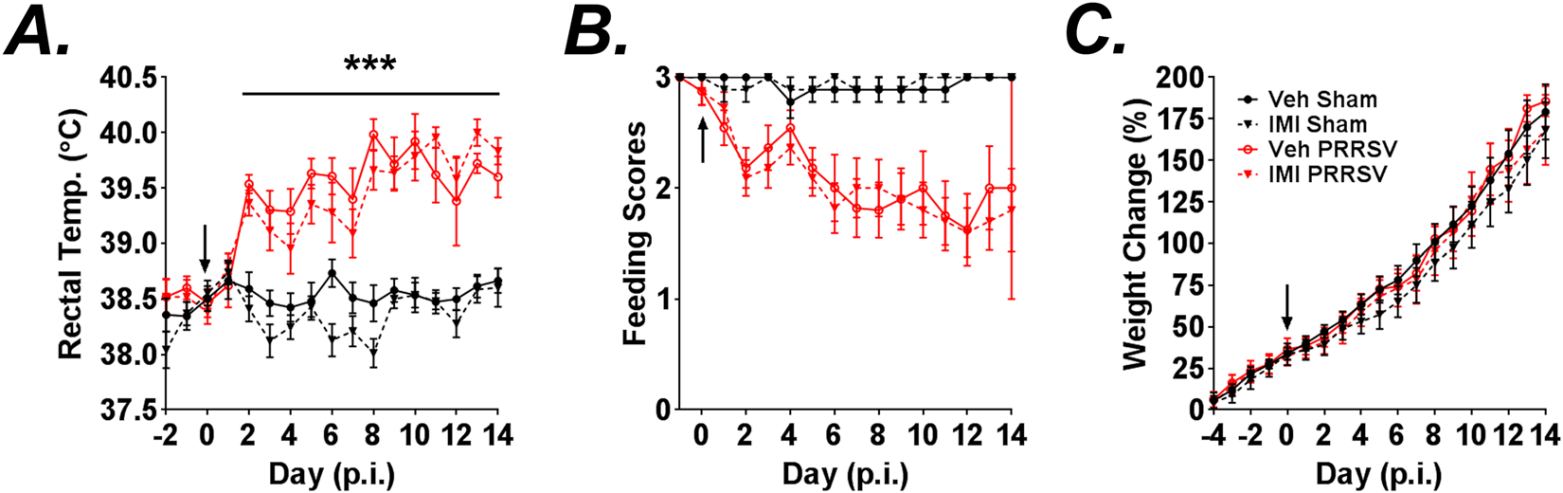
(**A**–**C**) Effect of infection and dietary imidacloprid (IMI) on weight, temperature, and feeding score. Feeding scores were recorded to assess willingness of the piglets to consume their first daily meal using a scoring system (1 = no attempt to consume the milk; 2 = attempted to consume the milk, but did not finish within 1 min; 3 = consumed all of the milk within 1 min). Piglets were inoculated with sterile saline or PRRSV 1 × 10^5^ TCID_50_. One day prior to infection diets were supplemented with vehicle or IMI (5 mg/kg/day). Percent weight change (**A**), rectal temperatures (**B**), and feeding scores (**C**) are shown. Arrows indicate the day of infection. Infection caused intermittent fevers starting at day 2 p.i. (p.i.) as well as a tendency for treatment with IMI to reduce fever caused by PRRSV infection (p ≤ 0.0577). A main effect of IMI on body weight was observed over the course of the experiment. Results are combined means ± S.E. from two independent experiments n = 3–11 pigs/group.

Having established the receptor expression profile of α7 nAChR on porcine immune cells we next questioned whether imidacloprid, which signals to a α-BT sensitive insect nAChR, could decrease porcine immune responses during a viral challenge. During the course of study body weights, rectal temperatures and feeding scores for each piglet were measured daily and used as indices of sickness behavior. PRRSV infected pigs developed intermittent fevers beginning at day 2 p.i. and lasting until day 14 p.i. (Fig. 2A). Ingestion of IMI also reduced body temperature, an effect that was most prominent during the first week (Fig. 2A). Furthermore, there was a strong trend for IMI to reduce fever caused by PRRSV infection, but the effect did not reach statistical significance (p = 0.058; Fig. 2C). Regardless of treatment, the onset of fever corresponded well with an observed reduction in feeding score (Fig. 2B). Piglets that received dietary IMI displayed a reduction in body weight compared with piglets that received dietary supplementation of vehicle (Fig. 2C). In contrast, treatment had no effect on feeding scores (Fig. 2B). Together, these data indicate that PRRSV infection causes sickness behavior and fever, but also that dietary supplementation with IMI at 5 mg/kg/day reduced body weight and caused hypothermia.

**Figure 3:**
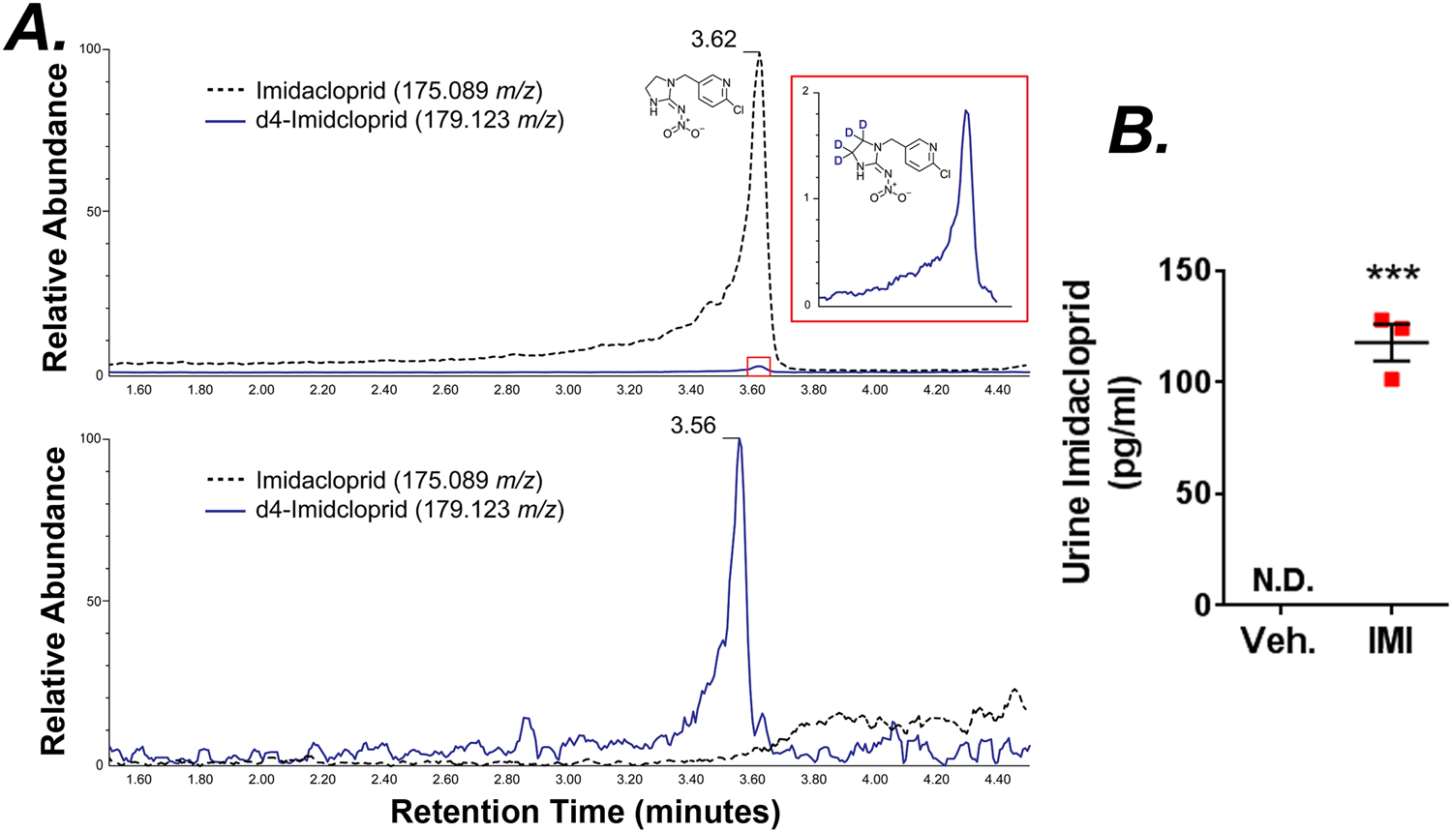
Urine imidacloprid was determined by LC-MS/MS. Representative and scaled LC-MS/MS MRM chromatographs showing the IMI levels in pig urine at day 14 alongside the d4-IMI internal standard after solid phase extraction. (**A**) Imidacloprid peak (quantification ion = 175.089 *m/z*) from IMI treatment urine shown with d4-imidacloprid peak (quantification ion = 179.123 *m/z*) in blue at a retention time of 3.62 minutes (*top*). Lack of imidacloprid peak from control (Veh.) urine shown with the detected d4-imidacloprid peak in blue at a retention time of 3.56 minutes (*bottom*). The variance in retention times is due to an insignificant column temperature change, although the quantification ions of 175.089 *m/z* and 179.123 *m/z* for imidacloprid and d4-imidacloprid, respectively, remain unchanged. (**B**) Quantification of urine IMI after normalizing for solid phase extraction using d4-imidacloprid spiked samples. N.D. is non-detectable. Results are means ± S.E. ***p < 0.001 by student’s t-test.

To confirm the absorption of neonicotinoids we measured urine IMI concentrations by mass spectroscopy. Importantly, mass spectroscopy analysis of urine samples taken at day 14 p.i. demonstrated the presence of IMI in the treatment group alone (Fig. 3A,B). The limit of quantification (LOQ) was determined to be 50 fg/μl with our system. Levels of urine imidacloprid from control pigs were below the limit of detection. In stark contrast, the concentration of IMI was substantially evaluated in treated pigs (Fig. 3B). These data clearly demonstrate the efficacy of our dietary treatment.

### Dietary imidacloprid increased serum IL-10 levels

**Figure 4:**
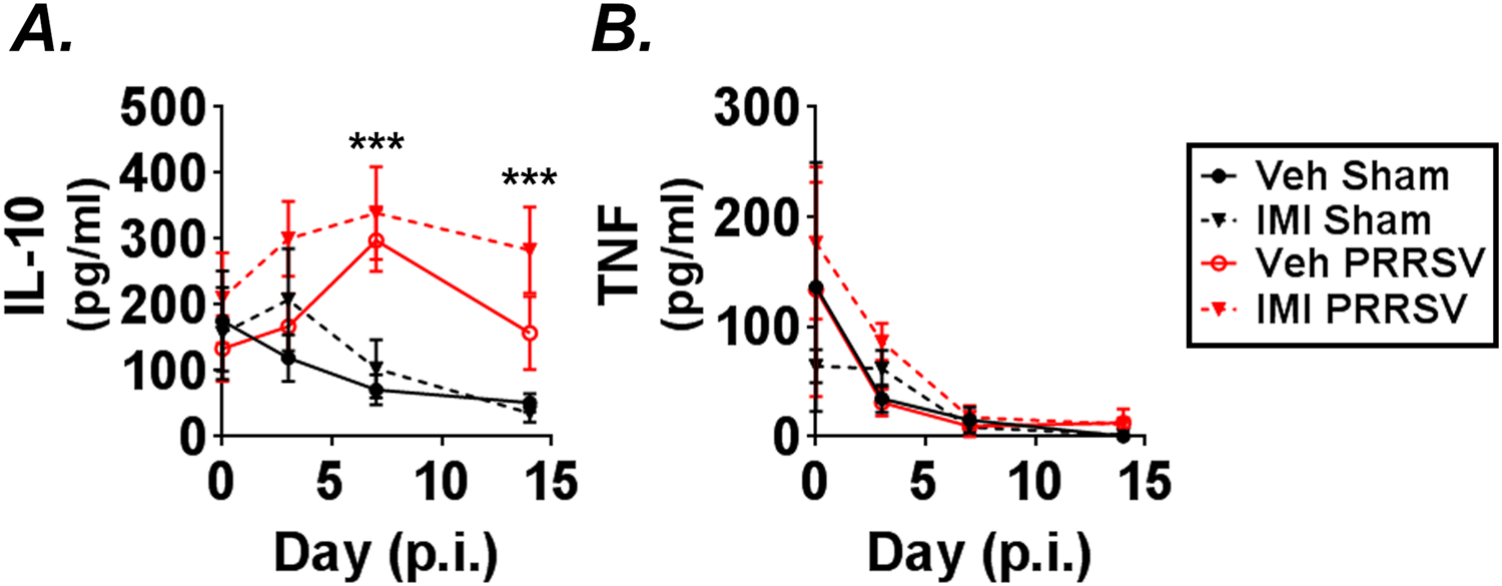
(**A**,**B**) Serum cytokines were measured on days 0, 3, 7, and 14 p.i. by ELISA. Effect of treatment and infection on serum IL-10 (**A**) and TNF (**B**) levels are shown. Imidacloprid (IMI) increased circulating IL-10 (p ≤ 0.01). Results are combined means ± S.E. from two independent experiments, ***p ≤ 0.001, Day 0 n = 4–10, Day 3 n = 7–9, Day 7 n = 7–10, Day 14 n = 7–10 pigs/group).

Serum concentrations of IL-10 and TNF were analyzed by ELISA. Infection increased serum levels of IL-10 at days 7 and 14 p.i. (Fig. 4A). Moreover, treatment with IMI increased circulating IL-10 in both PRRSV infected and non-infected groups (Fig. 4A). However, there was no significant interaction between PRRSV and IMI. Similarly, neither infection nor treatment altered the levels of serum TNF (Fig. 4B) or plasma IL-6 (Supplemental Fig. 3). These data indicate that both IMI treatment and infection with PRRSV increases circulating serum IL-10 levels.

### Effects of imidacloprid and PRRSV on circulating leukocytes

**Figure 5:**
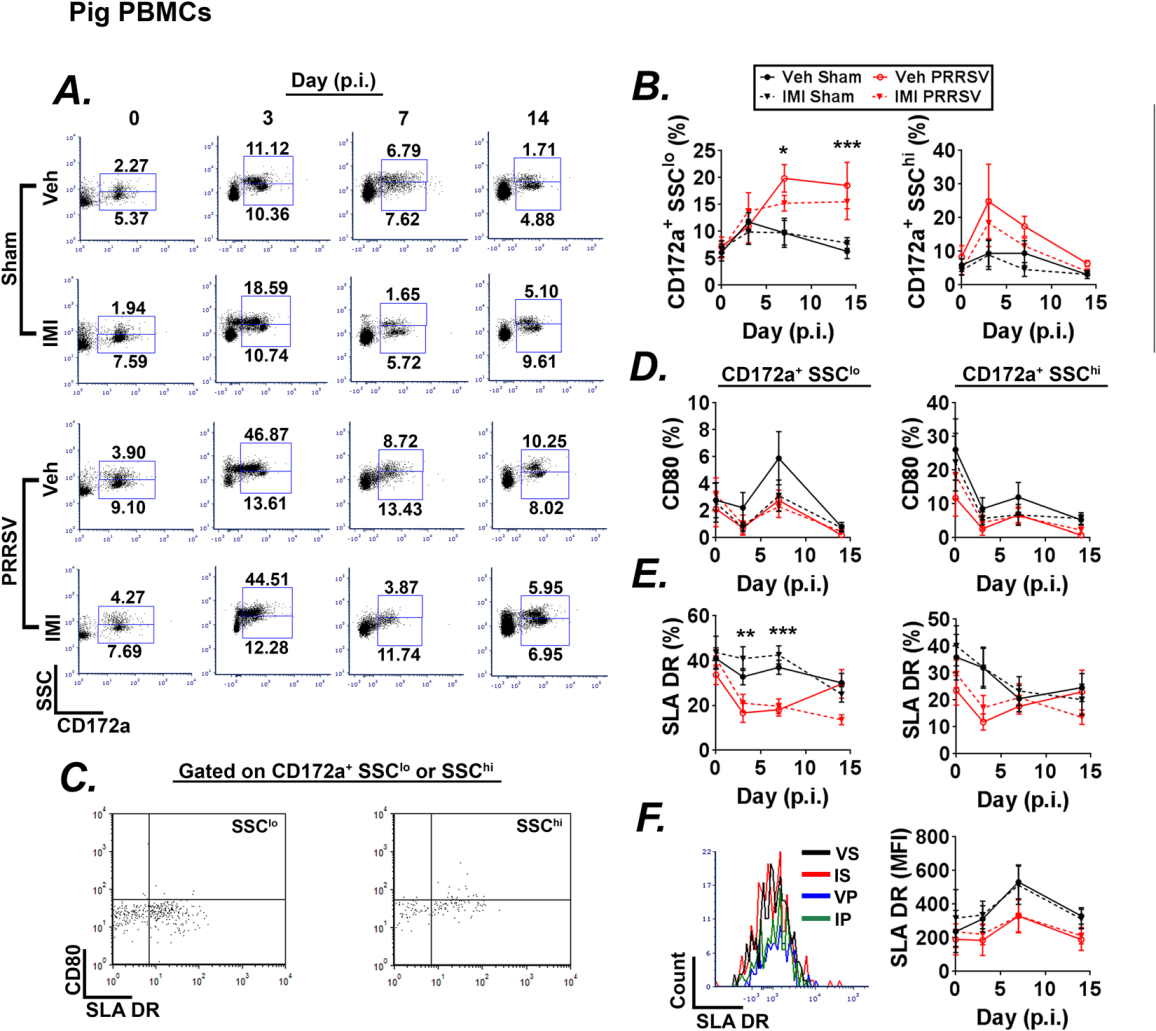
(**A**–**F**) Effect of infection and dietary imidacloprid (IMI) on peripheral blood leukocytes. Piglets were inoculated with sterile saline or PRRSV 1 × 10^5^ TCID_50_. One day prior to infection diets were supplemented with vehicle or IMI (5 mg/kg/day). The effect of both infection and treatment on leukocyte populations were determined at days 0, 3, 7, and 14 p.i. (**A**) Flow cytometry gating strategy for monocyte (CD172a^+^SSC^lo^) and granulocyte (CD172a^+^SSC^hi^) cells for all groups at days 0, 3, 7, and 14 p.i. (**B**) Percentages of CD172a^+^SSC^lo^ monocytes (*Left*) and CD172a^+^SSC^hi^ granulocytes (*Right*) at days 0, 3, 7, and 14 p.i. (**C**) Flow cytometry gating strategy used to evaluate surface expression and percentage of CD80^+^SLA DR^+^ cells on CD172a^+^SSC^lo^ monocytes (*Left*) and CD172a^+^SSC^hi^ granulocytes (*Right*). (**D**) Percentages of CD80 on CD172a^+^SSC^lo^ monocytes (*Left*) and CD172a^+^SSC^hi^ granulocytes (*Right*) at days 0, 3, 7, and 14 p.i. (**E**) Percentages of SLA DR^+^ gated cells on CD172a^+^SSC^lo^ monocytes (*Left*) and CD172a^+^SSC^hi^ granulocytes (*Right*) at days 0, 3, 7, and 14 p.i. Expression (*Left*) and mean fluorescent intensity (MFI; *Right*) of SLA DR on CD172a^+^SSC^lo^ monocytes at day 7 p.i. Results in (B–**F**) are combined means ± S.E. from two independent experiments, *p < 0.05, **p < 0.01, ***p < 0.001, Day 0 n = 6–11, Day 3 n = 5–9, Day 7 n = 8–11, Day 14 n = 7–10 pigs/group n = 9–10 pigs/group).

Next, we questioned whether IMI treatment was capable of altering the percentages of circulating immune cells during the course of acute PRRSV infection. Flow cytometric analysis was used to determine the effects of infection and dietary IMI on monocyte and granulocyte populations in blood at days 0, 3, 7, 14 p.i. (Fig. 5A). Infection increased circulating monocytes (CD172a^+^SSC^lo^) at days 7 and 14 p.i. (Fig. 5B). In contrast, the percentage of granulocytes (CD172a^+^SSC^hi^) was highest at day 3 p.i. but quickly returned to baseline levels (Fig. 5B). Treatment with IMI did not affect the percentages of circulating leukocytes.

In order to better understand the effect on infection and treatment on monocyte activation status, we measured the percentage of MHC class II (SLA DR)^+^ and CD80^+^ monocyte and granulocyte populations (Fig. 5C). The percentage of CD80^+^ monocyte and granulocyte populations did not change as a result of either infection or treatment (Fig. 5D). However, infection reduced the percentage of monocytes that expressed MHC class II at days 3 p.i. and 7 p.i. (Fig. 5E). At day 14 p.i. PRRSV infected piglets that received dietary supplementation with IMI appeared to have decreased percentages of MHC class II^+^ monocytes compared to controls, but the effect did not reach statistical significance (p = 0.06; Fig. 5E).

Eosinophils, basophils and mast cells also express surface MHC class II molecules and may be competent antigen presenting cells^35^. Therefore, we measured MHC class II expression on circulating granulocytes. The percentage of MHC class II^+^ granulocytes was reduced in infected piglets at day 3 p.i. (Fig. 5E).

In order to determine if the reduction in MHC class II^+^ cell monocytes from infected piglets was the result of decreased cell numbers or surface expression of these proteins, we analyzed the mean fluorescent intensity (MFI) of MHC class II on monocytes. Our results show PRRSV infected piglets had a lower MFI of MHC class II over the course of the experiment, indicating that the lower expression was likely result of a decrease in receptor levels per cell and not a decrease in cell number (Fig. 5F).

**Figure 6:**
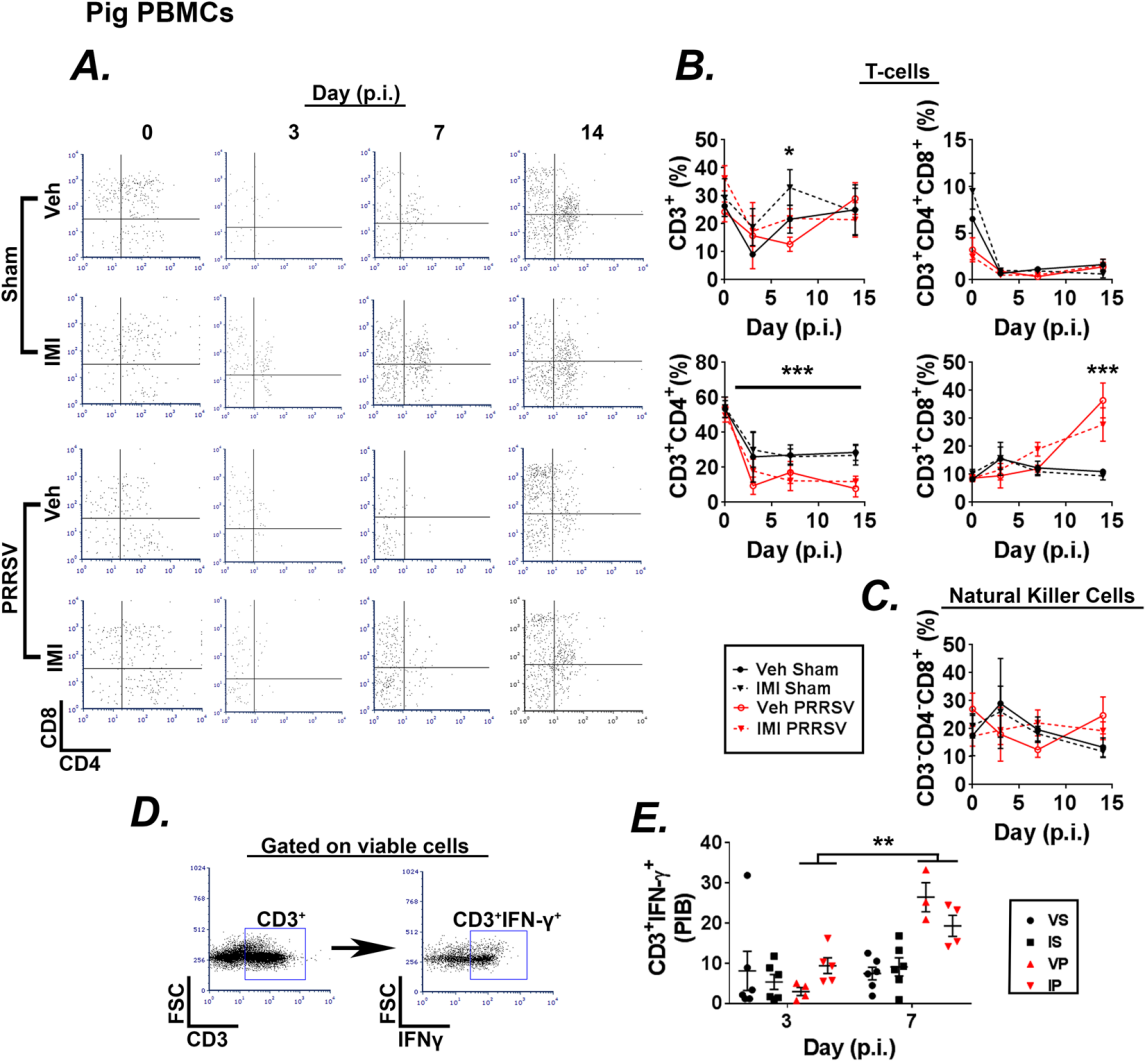
(**A**–**E**) Effect of infection and dietary imidacloprid (IMI) on circulating T-cells. Piglets were inoculated with sterile saline or PRRSV 1 × 10^5^ TCID_50_. One day prior to infection diets were supplemented with vehicle or IMI (5 mg/kg/day). Peripheral blood mononuclear cells (PBMCs) were analyzed at days 0, 3, 7, and 14 p.i. by flow cytometry. (**A**) Gating strategy used to determine helper (CD3^+^CD4^+^) and cytotoxic (CD3^+^CD8^+^) T-cells and natural killer cells (CD3^−^CD4^−^CD8^+^) for all groups at days 0, 3, 7, and 14 p.i. (**B**) Percentages of CD3^+^ (*Upper Left*; Main effect of imidacloprid, *p < 0.05), CD3^+^CD4^+^CD8^+^ (*Upper Right*), CD3^+^CD4^+^ (*Lower Left;* Main effects of time and infection, ***p’s < 0.001), and CD3^+^CD8^+^ (*Lower Right*) cells at days 0, 3, 7, and 14 p.i. (**C**) Percentages of CD3^−^CD4^−^CD8^+^ NK cells at days 0, 3, 7, and 14 p.i. (**D**,**E**) Following isolation PBMCs were stimulated with PMA and ionomycine in the presence of brefeldin-A for 4 h then stained for surface markers and intracellular IFN-γ. (**D**) Flow cytometry gating strategy for IFN-γ expression (*Right*) from T-cells (CD3^+^) (*Left*) for all groups at days 0, 3, 7, and 14 p.i. (**D**) Percentage of CD3^+^IFN-γ^+^ cell populations for all groups at days 3 and 7 p.i. are shown (**E**). Infection increased the percentage of CD3^+^IFN-γ^+^ cells at day 7 compared with day 3. Results in (**B**,**C** and **E**) are means ± S.E. from two independent experiments, *p < 0.05, ***p < 0.001, Day 0 n = 5–9, Day 3 n = 2–5, Day 7 n = 7–10, Day 14 n = 7–10 pigs/group.

Analysis of circulating lymphocyte populations suggest that infection reduced the percentage of circulating CD3^+^ T-cells at day 7 p.i. (Fig. 6B). Infection decreased circulating CD3^+^CD4^+^ T-cells throughout the course of the experiment (Fig. 6B). In contrast, the percentage of cytotoxic T-cells (CD3^+^CD8^+^) were elevated in infected piglets at day 14 p.i. (Fig. 6B). Infection did not affect the percentage of double positive memory T-cell (CD3^+^CD4^+^ CD8^+^) or NK cell (CD3^−^CD8^+^) populations (Fig. 6B,C). Treatment with IMI had no effect on circulating T-cell or NK cell populations.

The percentage of IFN-γ producing T-cells was analyzed by intracellular cytokine staining and flow cytometry (Fig. 6D). Infection increased the percentage CD3^+^IFN-γ^+^ cells at day 7 p.i. compared with day 3 p.i. (Fig. 6E). Furthermore, treatment with IMI appeared to reduce the percentage of percentage CD3^+^IFN-γ^+^ cells in the circulation at day 7 p.i., but this effect did not reach statistical significance (Fig. 6E). Collectively, these data indicate that dietary IMI has little, if any, effect on the percentage and effector function of circulating leukocytes during PRRSV infection.

### The effect of dietary imidacloprid on PRRSV-specific antibody levels

**Figure 7:**
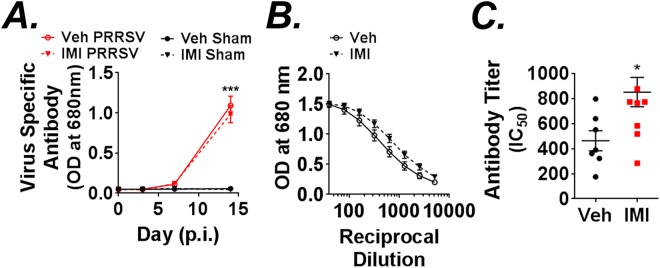
(**A**–**C**) Effect of dietary imidacloprid (IMI) and infection on virus-specific antibody levels. (**A**) Porcine reproductive and respiratory syndrome virus (PRRSV) specific antibody levels were analyzed by ELISA at days 0, 3, 7, and 14 p.i. (**B**,**C**) Dilution curve (**B**) and antibody titer (IC_50_; C) were generated following 2-fold serial dilution of serum obtained from infected piglets (*day 14*). Top dilutions started at a 1:40 dilution and bottom dilution was 1:5120. Results in (**A**–**C**) are means ± S.E. from two independent experiments. D0, n = 4–9; D3, n = 7–9; D7, n = 7–10; D14, n = 7–10 per group. *p < 0.05, ***p < 0.001.

In order to determine the ability for dietary imidacloprid to effect the generation of virus-specific immunity we measured virus-specific antibodies levels in the plasma. Infected piglets developed a small, but detectible, antibody response at day 7 p.i. This response was greatly increased at day 14 p.i. In contrast, virus-specific antibody was not detectable in non-infected piglets (Fig. 7A). Within infected groups, the antibody level was saturated at a dilution of 1/40 at day 14 p.i. Therefore, in order to better analyze antibody levels between the PRRSV infected groups serial dilutions of plasma were analyzed. Interestingly, piglets that received dietary supplementation of IMI had higher levels of circulating antibody than vehicle control piglets (Fig. 7B), which was also evident after measuring the antibody titer (Fig. 7C).

### Effects of dietary imidacloprid on PRRSV dissemination

**Figure 8:**
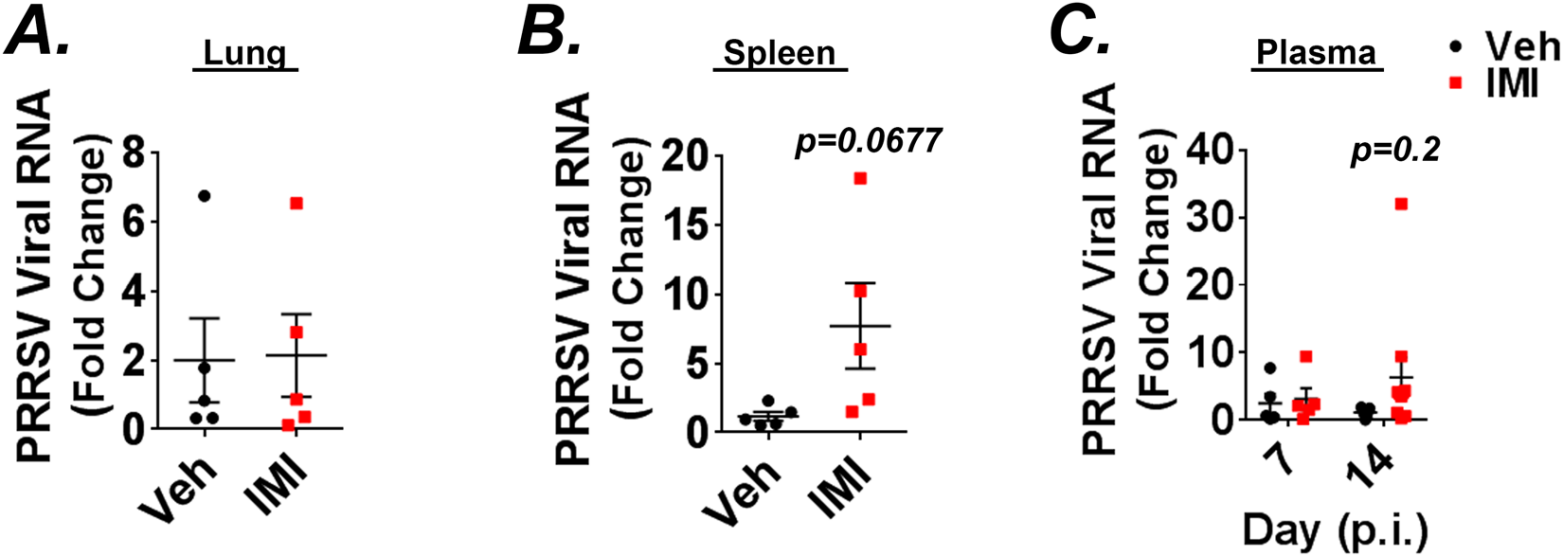
(**A**–**C**) Effect of imidacloprid (IMI) on viral RNA levels in lung, spleen and plasma. Porcine tissues from infected piglets treated with vehicle or IMI (5 mg/kg/day) were analyzed for the presence of PRRSV RNA by RT-qPCR using the formula 2^−∆Ct^. (**A**,**B**) Effect of treatment on viral RNA expression in the lung (**A**) and spleen (**B**) at day 14 p.i. (n = 5 pigs/group). (**C**) Viral RNA expression in the plasma at days 7 and 14 p.i. normalized to vehicle day 14 p.i. samples. Results in (**A**–**C**) are means ± S.E. Results in (**A**,**B**) are from one experiment and results in (**C**) are combined means from two independent experiments n = 5–10 pigs/group.

Given the effect of IMI treatment on the generation of virus-specific antibody levels, we questioned whether dietary IMI could alter the viral RNA levels in different tissues. The lung is the primary site of PRRSV replication, but virus can also be detected in lymphoid tissues including regional lymph nodes, thymus, and spleen. Therefore, lung, blood and spleen tissue samples were collected for viral RNA determination by real-time PCR. Our results show that while viral RNA was undetectable in the lung of sham inoculated control piglets, it was highly expressed in lung biopsies obtained from infected piglets at day 14 p.i. (Supplemental Fig. 4). However, lung viral RNA levels in infected piglets were almost identical between treatment groups at this time-point (Fig. 8A). In contrast, viral RNA levels from piglets supplemented with IMI showed a strong trend toward being increased in the spleen (p = 0.0677; Fig. 8A,B), indicating that treatment may have cause increased viremia and viral dissemination. Based on these data we next analyzed the effect IMI on viral RNA in the plasma of infected piglets at days 7 and 14 p.i. No differences were detectable between vehicle and IMI groups at day 7 p.i. However, at day 14 p.i. piglets supplemented with IMI appeared to have increased levels of viral RNA although the effect did not reach statistical significance (Fig. 8C). Together, these data suggest that dietary supplementation with IMI may promote viremia and dissemination of PRRSV.

## Discussion

In the current study we used a porcine model to investigate the potential for dietary imidacloprid treatment to alter the generation of immune responses during viral infection. Importantly, we detected IMI in the urine of piglets in the treatment group, but not in the vehicle control group. We found that IMI treatment caused bouts of hypothermia that were independent of infection status. Treatment also reduced body weight, an effect that was most prominent in infected piglets. Additionally, we showed that dietary IMI slightly increased circulating levels of IL-10. Finally, our data also indicates that IMI treatment during PRRSV infection may increase virus-specific antibody production, but also may promote viremia and viral dissemination. To our knowledge this study is the first to investigate the effects of IMI on the generation of pathogen-specific immunity and sickness behaviors using a mammalian model.

It is well established that nicotine can dose-dependently induce a transient hypothermic state. Nicotine-induced hypothermia can be blocked by treatment with mecamylamine and dihydro-β-erythroidine, but not methyllycaconitine, indicating that this physiologic response to nicotine is attributable to its ability to activate α4β2 nAChRs rather than the α7 nAChR^36^. Mammalian body temperature is tightly regulated by the hypothalamus. That dietary IMI induced hypothermia in our experiments is intriguing and may suggest that it is capable of activating hypothalamic α4β2 nAChRs. Indeed, biochemical analysis has shown that IMI binds the mammalian α4β2 nAChR with greater affinity than the α7 nAChR^37^. While it has been suggested that IMI does not readily cross the blood brain barrier^37^, recent experiments conducted in rats indicate that IMI and two of its major metabolites, 6-hydroxy nicotinic acid and 6-chloronicotinic acid, are detectable in the brain twelve hours after receiving a single oral dose of 20 mg/kg^16^. Notably this dose exceeds the No Observable Adverse Effect Level (NOAEL) for rats. It is therefore not surprising that the authors of this study observed salivation, diarrhea, dyspnea and piloerection in treated animals. However, given that we observed hypothermia, it is notable that the authors of the above study observed tremors at 6 and 12 hours post treatment^16^ which corresponded to peak brain concentrations. Unfortunately, body temperature in this study was not reported.

Infection with PRRSV increased serum levels of IL-10. We also found that dietary IMI slightly increased circulating IL-10 in a manner that was independent of infection status. The vagal nerve acts as a regulator of immunity and immunosuppression brought on by either ACh or nicotine stimulation of α7 nAChR is well established^38^. However, the ability for either ACh or nicotine to stimulate production of anti-inflammatory cytokines by immune cells is not as clear. For instance, Borovikova *et al*., have provided compelling evidence that suggests IL-10 production by macrophages is not affected by ACh treatment and that vegotomy did not alter serum IL-10 levels following a peripheral challenge with LPS^23^. Similarly, it was reported that nicotine stimulation did not affect IL-10 production from cultured splenocytes *ex vivo*^39^. In contrast, nicotine stimulation of peritoneal macrophages dose-dependently inhibited TNF and CCL2 production, but increased expression of IL-10^40^. In this study, the effect of nicotine on IL-10 production was only significantly increased when the intracellular protein was measured, indicating that assay sensitivity may underlie the discrepancies between studies. It is unclear at this time whether imidacloprid, or any of its metabolites, are capable of acting on either the α4β2 or the α7 nACh to promote IL-10 production or if the small increase in circulating IL-10 that we observed is biologically relevant. Culture experiments designed to investigate the effect of treatment by imidacloprid or its metabolites on IL-10 production from porcine B-cells, macrophages or dendritic cells may shed some light on this capacity.

One of the major findings from the current set of experiments is that dietary imidacloprid increased circulating antibody levels against PRRSV. It appeared that treatment also concomitantly increased viral dissemination. The effects of high dose IMI treatment on circulating antibody levels has been previously reported in one other study using male albino rats. In these studies IMI treatment with a dose that was determined be the LD_50_ increased circulating immunoglobulin^41^. Our data suggests that a much lower dose of IMI can modulate virus-specific antibody levels. The ability for IMI to increase humoral immune responses as well as elevate viral RNA levels might first appear contradictory. However, we did not evaluate the effect of treatment on neutralizing antibody levels. The discrepancy between neutralizing and non-neutralizing antibody levels is relevant since a large proportion of circulating antibody to PRRSV is non-neutralizing. Several viruses, most famously dengue virus, use non-neutralizing antibody to facilitate Fc-receptor mediated infection. Similarly, rather than conferring protection, non-neutralizing PRRSV-specific antibodies promote viral dissemination through the process of antibody-dependent enhancement involving both FcγRII (CD32) and FcγRIII (CD16) mediated uptake^42–44^. It is therefore tempting to speculate that in our model imidacloprid treatment increased non-neutralizing virus-specific antibody titers which encouraged infection of macrophages and increased viral RNA levels in the spleen. This hypothesis has yet to be tested.

Finally, it is important to consider the dose of IMI used for these experiments, especially when attempting to draw conclusions from these data. In our study piglets received a daily dose of 5 mg/kg/d of IMI. Since the IMI we used was 95% pure, each pig in the treated group consumed 4.7 mg/kg/d. The NOAEL in rats is 5.7 mg/kg^45^. When calculating an acceptable daily intake value for humans the lowest observed NOAEL value obtained from toxicological experiments conducted in animals is divided by the safety factor (SF) which is usually set to a value of 100. This value is known as the chronic reference dose (RfD), or maximal daily dose that can be consumed for an entire lifetime. Based on the NOAEL obtained in rats the reference dose for IMI consumption in humans, is calculated to be 0.057 mg/kg/d (57 µg/kg/d). Recently, human daily intake values of neonicotinoids were predicted based on toxicological modeling by a study conducted in Japan. These data indicated that the average adult IMI consumption is approximately 100 fold less IMI than the reference dose set by U.S. Environmental Protection Agency. Daily consumption values of other neonicotinoids, including dinotefuran (3.66 µg/d), acetamiprid (1.94 µg/d), and clothianidin (0.86 µg/d) were also predicted. The NOAEL for IMI has not been determined in piglets. While we did not examine all of the potential toxicological effects of IMI in piglets, our data indicate that 4.7 mg/kg/d is capable of altering physiological responses in this animal model system. Nevertheless, caution should be used when interpreting these results. Another caveat of the current experiment is that we did not examine the effects of imidacloprid treatment on mucosal immunity, especially given the route by which IMI was administered.

In conclusion, our data suggests that dietary supplementation of IMI during PRRSV infection of piglets does not alter the percentage of circulating leukocytes. However, daily treatment affected rectal temperatures and body weights in piglets. Furthermore, IMI treatment increased circulating IL-10 levels, virus-specific antibody titers and showed a statistical trend toward promoting PRRSV dissemination.

## Electronic supplementary material

Supplemental Figures
