# Supplementary material for: Effect of imidacloprid ingestion on immune responses to porcine reproductive and respiratory syndrome virus
Source: Sci Rep. 2018 Aug 2;8:11615. doi: 10.1038/s41598-018-30093-6 (PMC6072715; doi:10.1038/s41598-018-30093-6)
Supplement: Supplementary file 1 — Supplemental Figures [file 41598_2018_30093_MOESM1_ESM.pdf]

Supplemental Figures and Legends:

Effect of imidacloprid ingestion on immune responses to porcine reproductive and respiratory syndrome virus

Hernandez J.[1,2], Volland A.[1], Leyshon B.J.[4], Juda M.[1], Ridlon, J.[1,3,4], Johnson R.W.[1,2,4,5], Steelman A.J.[1,2,4,5,*].

[1]Department of Animal Sciences, [2]Integrative Immunology and Behavior Program, [3]Carl R. Woese Institute for Genome Biology, [4]Division of Nutritional Sciences, [5]Department of Neuroscience, University of Illinois Urbana-Champaign, Urbana, IL 61801.

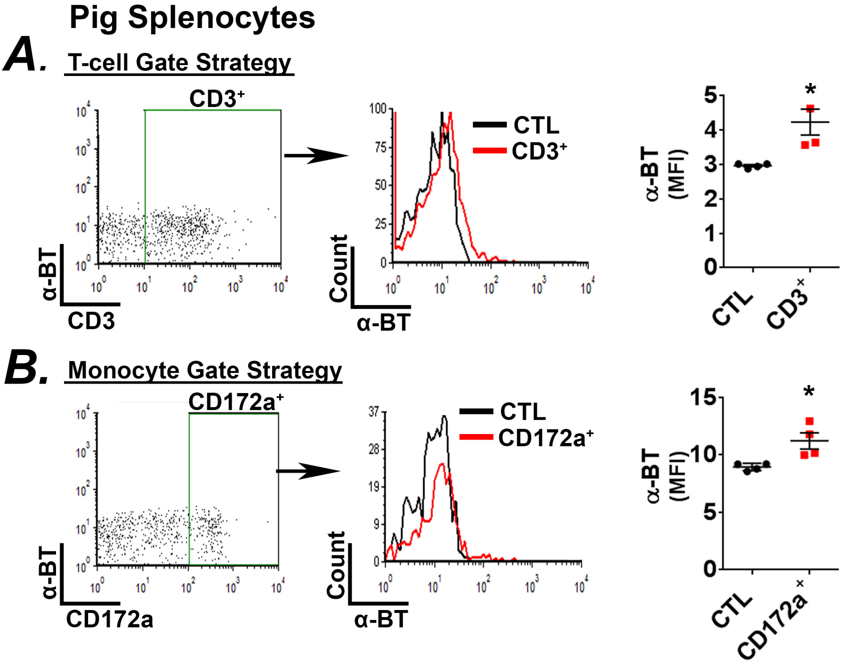
Pig Splenocytes
A.
T-cell Gate Strategy
CD3+
α-BT
CD3
Count
α-BT
CTL
CD3+
α-BT (MFI)
*
CTL
CD3+
B.
Monocyte Gate Strategy
CD172a+
α-BT
CD172a
Count
α-BT
CTL
CD172a+
α-BT (MFI)
*
CTL
CD172a+

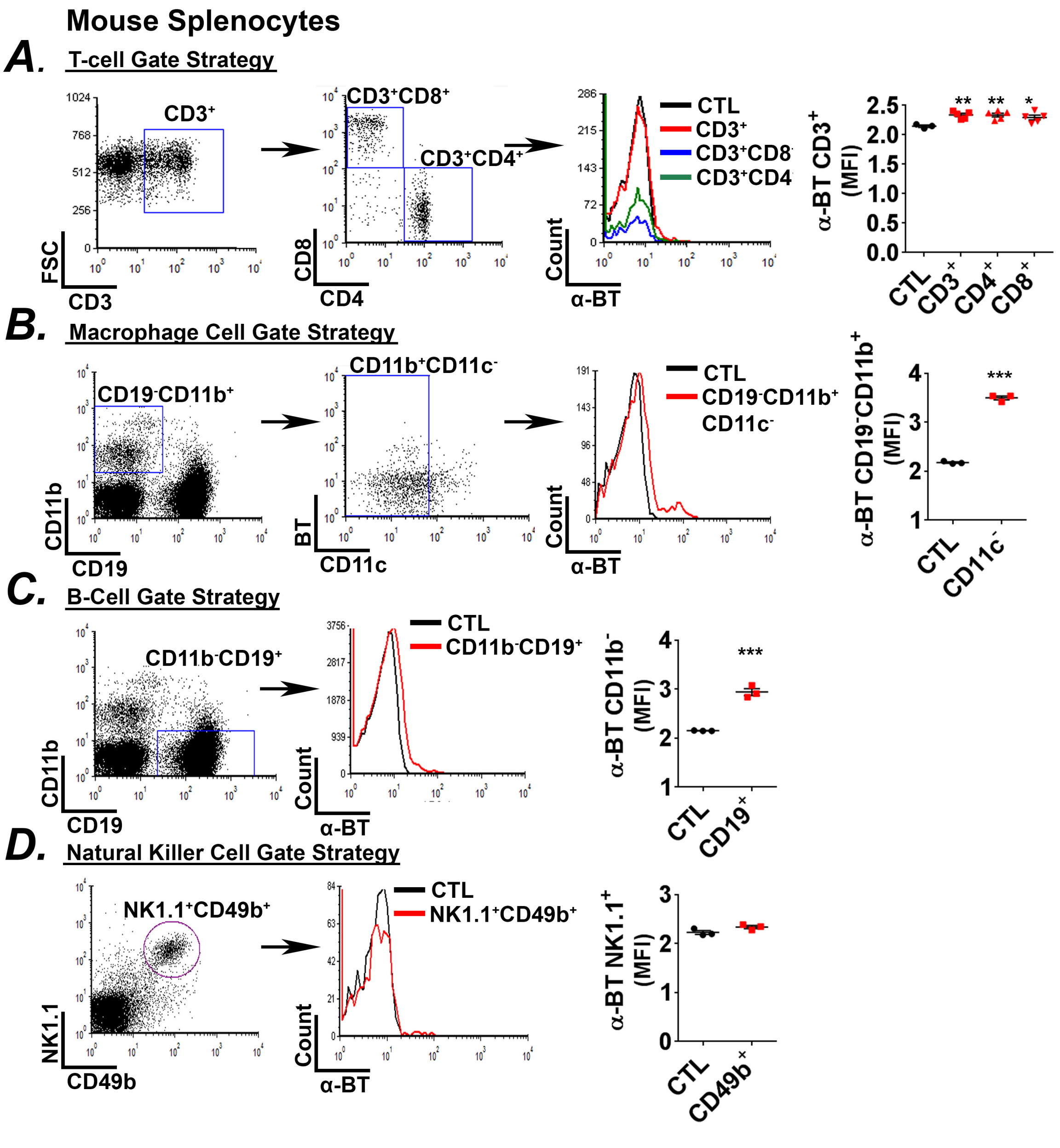
Mouse Splenocytes
A. T-cell Gate Strategy
CD3+
FSC
CD3
CD3+CD8+
CD3+CD4+
CD8
CD4
CTL
CD3+
CD3+CD8
CD3+CD4
Count
α-BT
α-BT CD3+ (MFI)
CTL
CD3+
CD4+
CD8+
B. Macrophage Cell Gate Strategy
CD19-CD11b+
CD11b
CD19
CD11b+CD11c-
BT
CD11c
CTL
CD19-CD11b+ CD11c-
Count
α-BT
α-BT CD19-CD11b+ (MFI)
CTL
CD11c-
C. B-Cell Gate Strategy
CD11b-CD19+
CD11b
CD19
CTL
CD11b-CD19+
Count
α-BT
α-BT CD11b- (MFI)
CTL
CD19+
D. Natural Killer Cell Gate Strategy
NK1.1+CD49b+
NK1.1
CD49b
CTL
NK1.1+CD49b+
Count
α-BT
α-BT NK1.1+ (MFI)
CTL
CD49b+

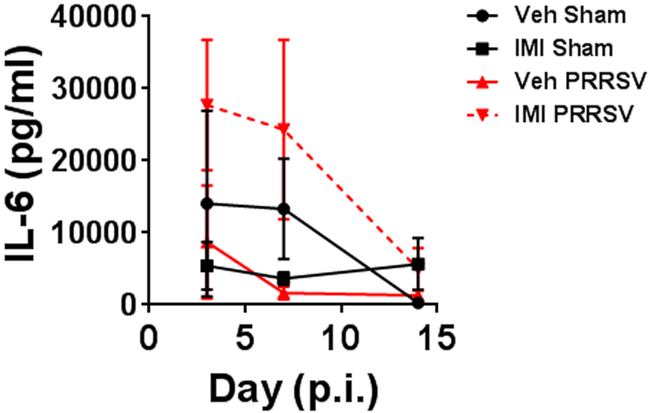

IL-6 (pg/ml)
40000
30000
20000
10000
0
0
5
10
15
Day (p.i.)
Veh Sham
IMI Sham
Veh PRRSV
IMI PRRSV

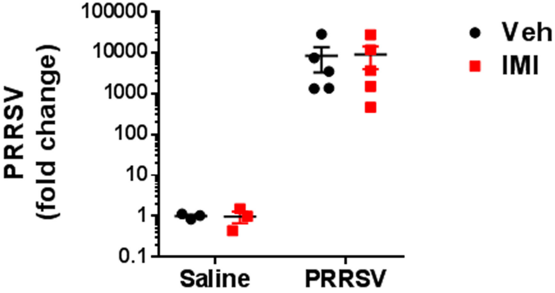
PRRSV
(fold change)
100000
10000
1000
100
10
1
0.1
Saline
PRRSV
Veh
IMI

Supplemental Figure Legends

Supplemental Figure 1: ***A-B,*** The expression of the α7 nicotinic acetylcholine receptor (α7nAChR) on splenic T-cells ($CD3^{+}$) (***A***) and monocytes ($CD172a^{+}$) (***B***) was analyzed by flow cytometry after staining with, fluorescently labeled α-bungarotoxin (α-BT). Flow cytometry gating strategy is provided for each cell type (*Left*), histograms show representative expression between unstained and α-BT stained cells (*Middle*), and graphs represent mean fluorescent intensity (MFI) of α-BT between unstained controls and α-BT stained cells (*Right*). Results are combined means ± S.E. from 4 pigs per group. *p≤0.05.

Supplemental Figure 2: ***A-D,*** The surface expression pattern of the α7 nicotinic acetylcholine receptor (α7nAChR) on C57BL/6 mouse splenocytes was analyzed for their ability to bind fluorescently labeled α-bungarotoxin (α-BT) on T-cells (***A***; n= 3-5), macrophages (***B***; n= 3), B-cells (***C***; n= 3), and natural killer cells (***D***; n= 3). Flow cytometry gating strategy is provided for each cell type (*Left*), histograms show representative expression between unstained controls and α-BT stained cells (*Middle*), and graphs (*Right*) represent mean fluorescent intensity (MFI) of α-BT between unstained controls and α-BT stained cells. Results are means ± S.E. from 3-8 pigs. *p≤0.05, ***p≤0.001.

Supplemental Figure 3: Plasma IL-6 levels on days 3, 7, and 14 p.i. as determined by ELISA. Results are combined means ± S.E. from two independent experiments, Day 3 n=4-10, Day 7 n= 3-6, Day 14 n=4-11 pigs/group).

Supplemental Figure 4: Effect of imidacloprid (IMI) and infection on viral RNA levels in lung. Lung biopsies from non-infected and infected piglets treated with vehicle or IMI (5 mg/kg/day) were analyzed for the presence of PRRSV RNA by RT-qPCR using the formula $2^{-\Delta Ct}$. Expression data is normalized to non-infected vehicle treated group. Results are means ± S.E. n=5 per group. *$p \leq 0.05$, ***$p \leq 0.001$.
